# Supplementary material for: mtDNA “nomenclutter” and its consequences on the interpretation of genetic data
Source: BMC Ecol Evol. 2024 Aug 19;24:110. doi: 10.1186/s12862-024-02288-1 (PMC11331612; doi:10.1186/s12862-024-02288-1)
Supplement: Supplementary file 1 — Supplementary Material 1: Table S1.The number of haplogroup names from phylotree-fu-rcrs-1.2that do not follow strict cladistic nomenclature with haplogroup names starting with capital letters followed with alternating numbers and small letters. Note that the “cladistic notation for mitochondrial clusters” established by Richards et al. 1998permits composed names to refer to monophyletic clades based on subclades, and usage of * that designates all sequences that do not belong to any of the already named subclades. Table S2. Overview of Nomenclature-Based Groupingsused in the research articles published in years 2020-2023 through Nature Publishing Groupwith the search term: “africa* haplogrep* human* mtdna* haplogroup* OR africa* haplogrep* human* mitochondria* haplogroup*”. Each research article was categorized based on the NBG employed. Available at GitHub: https://github.com/bajicv/mtDNA_nomenclutter/blob/main/supplements/Table_S2_mtDNA_Nomenclutter.xlsx and at Zenodo: https://doi.org/10.5281/zenodo.10156923. Figure S1. The mtDNA ML consensus tree of 660 samples belonging to seven African Ancestry populationsfrom the Phase 3 release of the 1KGP. Colors indicate SCL membership. RSRSwas added for rooting the tree. The labels indicate haplogroup reported by HaploGrep3. Figure S2. MDS plots based on the mtDNA pairwise distances between individuals colored by NBGs: A) SC, and B) SCL; and ABGs: C-E) rhierBAPS, and F-I) TreeCluster. Note remarkable differences between SC and SCL as well as an increase in the number of haplogroups with increasing threshold values for ABGs. [file 12862_2024_2288_MOESM1_ESM.docx]

# Supplementary Material

## Supplementary Tables

| **[ Symbol ]** | **No.** | **%** | **Example** | **Comment** |
| --- | --- | --- | --- | --- |
| **[ - ]** | **1** | 0,02% | mt-MRCA |  |
| **[ . ]** | **1** | 0,02% | R0a+60.1T | Insertions are indicated by the position number preceding the insertion followed by a dot (.), the relative insert position, and the inserted base(s) (from PhyloTree.org - mtDNA tree Build 17). |
| **[ " ]** | **2** | 0,03% | M4"67+16311 | (as M4&quot;67 in tree.xml) |
| **[ ( ] and [ ) ]** | **31** | 0,49% | M7b1a1+(16192) | (always occurs together with +) Mutations between brackets () are recurrent/unstable within the respective clade, or are yet uncertain based on current data (from PhyloTree.org - mtDNA tree Build 17). |
| **[ @ ]** | **34** | 0,53% | U5a1+@16192 |  |
| **[ ' ]** | **109** | 1,71% | U2c'd |  |
| **[A-Z][A-Z]** | **116** | 1,82% | CZ, HV, JT, R2'JT | (excluding mt-MRCA)  A clade can be referred to using the names of its prominent subclades. E.g., CZ refers to the smallest monophyletic clade including Y and Z (Richards et al. 1998). |
| **[a-z][a-z]** | **179** | 2,81% | A2aa | (excluding mt-MRCA) |
| **[ + ]** | **305** | 4,78% | U2+152 |  |
| **[ * ]** | **945** | 14,81% | U6b* | Haplogroup followed by * denotes all sequences outside of its already named clades.  E.g., if U6b1, U6b2, and U6b3 are named then:  U6b* = U6b - ( U6b1 ∪ U6b2 ∪U6b3).  Such derived cluster notations are only preliminary and may change in the future (Richards et al. 1998). |
| **at least one of [*()@+"]** | **1199** | 18,79% | V+@72,  U5b1+16189+@16192 |  |
| **at least one of ['*-()@+"]** | **1306** | 20,47% | X2+225+@153, U6a+16189+(103), U3a'c | (including mt-MRCA) |
| **at least one exception** | **1559** | 24,44% | A2ae*2, HV+16311*, X2m'n |  |
| **without exception** | **4821** | 75,56% | X2e2a1 |  |

**Table S1.** The number of haplogroup names from *phylotree-fu-rcrs-1.2* (total of 6380 haplogroups) that do not follow strict cladistic nomenclature with haplogroup names starting with capital letters followed with alternating numbers and small letters. Note that the “cladistic notation for mitochondrial clusters” established by Richards et al. 1998 (see Test Box 1) permits composed names to refer to monophyletic clades based on subclades (e.g., CZ), and usage of * that designates all sequences that do not belong to any of the already named subclades (green rows in the table).

**Table S2.** Overview of Nomenclature-Based Groupings (NBGs) used in the research articles published in years 2020-2023 through Nature Publishing Group (https://www.nature.com/search/advanced) with the search term: “africa* haplogrep* human* mtdna* haplogroup* OR africa* haplogrep* human* mitochondria* haplogroup*”. Each research article was categorized based on the NBG employed. Available at GitHub: <https://github.com/bajicv/mtDNA_nomenclutter/blob/main/supplements/Table_S2_mtDNA_Nomenclutter.xlsx> and at Zenodo: <https://doi.org/10.5281/zenodo.10156923>.

|  | **n** | **NBG** | | **ABG** | | | | | | |
| --- | --- | --- | --- | --- | --- | --- | --- | --- | --- | --- |
|  |  |  |  | **rhierBAPS** | | | **TreeCluster** | | | |
|  |  | **SC** | **SCL** | **rhb_01** | **rhb_02** | **rhb_03** | **tc_0.006** | **tc_0.005** | **tc_0.004** | **tc_0.003** |
| J2b1a | 1 | J | J | 1 | 1 | 1 | 1 | 1 | 48 | 48 |
| U6a | 10 | U | U | 1 | 1 | 1 | 1 | 1 | 48 | 48 |
| B4a1a1b | 1 | B | B | 1 | 1 | 1 | 1 | 1 | 48 | 48 |
| H7 | 1 | H | H | 1 | 1 | 1 | 1 | 1 | 48 | 48 |
| A2 | 2 | A | A | 1 | 1 | 1 | 1 | 1 | 48 | 63 |
| C | 2 | C | C | 1 | 1 | 1 | 1 | 1 | 1 | 1 |
| D1 | 1 | D | D | 1 | 1 | 1 | 1 | 1 | 1 | 1 |
| M | 2 | M | M | 1 | 1 | 1 | 1 | 1 | 1 | 1 |
| L3h1a | 4 | L | L3 | 1 | 1 | 1 | 1 | 1 | 1 | 67 |
| L3h1b | 8 | L | L3 | 1 | 1 | 1 | 1 | 1 | 1 | 66 |
| L3b | 75 | L | L3 | 1 | 33 | 38 | 1 | 1 | 1 | 65 |
| L3d | 45 | L | L3 | 1 | 33 | 33 | 1 | 1 | 1 | 64 |
| L3a2 | 1 | L | L3 | 1 | 1 | 1 | 1 | 1 | 57 | 57 |
| L3k | 4 | L | L3 | 1 | 1 | 1 | 1 | 1 | 50 | 50 |
| L3e | 100 | L | L3 | 1 | 1 | 37 | 1 | 1 | 50 | 50 |
| L3f1b | 14 | L | L3 | 1 | 1 | 1 | 1 | 1 | 53 | 53 |
| L4b2 | 7 | L | L4 | 1 | 1 | 1 | 1 | 1 | 55 | 55 |
| L4b1 | 5 | L | L4 | 1 | 1 | 1 | 1 | 1 | 54 | 54 |
| L2a | 135 | L | L2 | 1 | 35 | 35 | 31 | 31 | 31 | 31 |
| L2a5 | 1 | L | L2 | 2 | 31 | 31 | 31 | 31 | 31 | 31 |
| L2c | 41 | L | L2 | 2 | 31 | 43 | 31 | 31 | 51 | 62 |
| L2b | 24 | L | L2 | 2 | 31 | 42 | 31 | 31 | 51 | 51 |
| L2d | 7 | L | L2 | 2 | 31 | 31 | 31 | 31 | 56 | 56 |
| L2e | 7 | L | L2 | 2 | 31 | 31 | 31 | 47 | 47 | 47 |
| L5b1 | 2 | L | L5 | 2 | 2 | 36 | 36 | 36 | 52 | 52 |
| L5a1 | 3 | L | L5 | 2 | 2 | 36 | 36 | 36 | 36 | 36 |
| L5b2 | 5 | L | L5 | 2 | 2 | 36 | 36 | 46 | 46 | 46 |
| L1c1c | 7 | L | L1 | 2 | 2 | 2 | 2 | 2 | 2 | 61 |
| L1c1a'b'd | 11 | L | L1 | 2 | 2 | 2 | 2 | 2 | 2 | 60 |
| L1c2 | 14 | L | L1 | 2 | 2 | 44 | 2 | 2 | 2 | 59 |
| L1c3 | 10 | L | L1 | 2 | 2 | 2 | 2 | 2 | 2 | 2 |
| L1b2 | 7 | L | L1 | 2 | 34 | 39 | 2 | 45 | 45 | 45 |
| L1b1a | 63 | L | L1 | 2 | 34 | 34 | 2 | 45 | 45 | 45 |
| L0f | 1 | L | L0 | 2 | 32 | 32 | 32 | 32 | 49 | 68 |
| L0f1 | 1 | L | L0 | 2 | 32 | 32 | 32 | 32 | 49 | 49 |
| L0b | 3 | L | L0 | 2 | 32 | 41 | 32 | 32 | 32 | 58 |
| L0a | 35 | L | L0 | 2 | 32 | 40 | 32 | 32 | 32 | 32 |
|  | **660** | **9** | **14** | **2** | **7** | **16** | **5** | **8** | **18** | **29** |
|  | **SUM** | **Number of groups** | | | | | | | | |

**Table S3.** Results of NBGs and ABGs used for plotting Figure 3. For exact correspondence between colors and names of groups see scripts/my_colors.R at <https://zenodo.org/records/10156923>

## Supplementary Figures


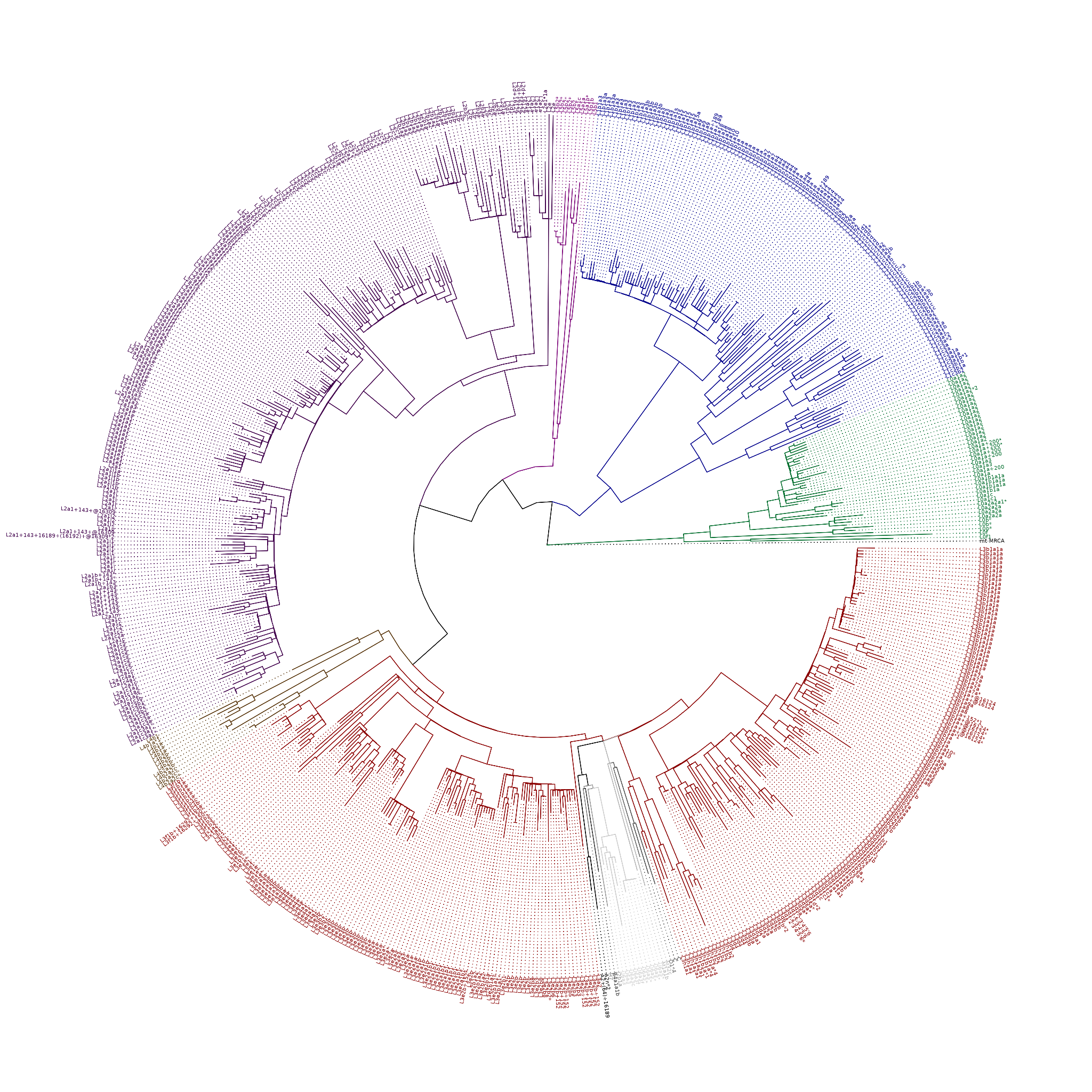


**Figure S1. The mtDNA ML consensus trees of 660 samples belonging to seven African Ancestry populations (ACB, ASW, MSL, GWD, ESN, YRI, LWK) from the Phase 3 release of the 1KGP.** Colors indicate SCL membership (single character and L with one-digit grouping). RSRS (mt-MRCA) was added for rooting the tree. The labels indicate haplogroup reported by *HaploGrep3*.


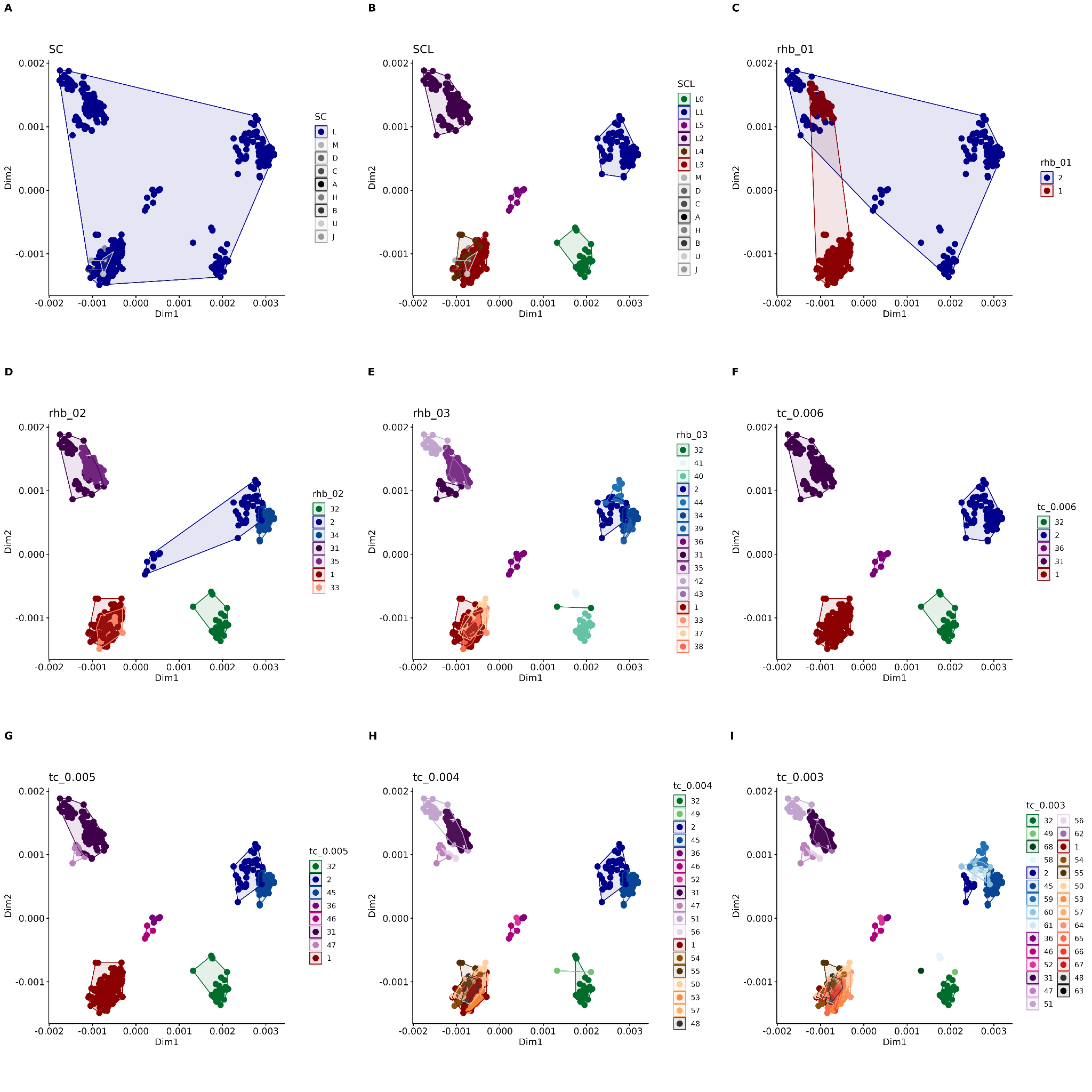


**Figure S2. MDS plots based on the mtDNA pairwise distances between individuals colored by NBGs: A) SC, and B) SCL; and ABGs: C-E) *rhierBAPS*, and F-I) *TreeCluster*.** Note remarkable differences between SC and SCL as well as an increase in the number of haplogroups with increasing threshold values for ABGs.
